# The Efficient Matron Who Creates Happiness

**Published:** 1919-02-01

**Authors:** Vera Matheson


### THE EFFICIENT MATRON WHO CREATES HAPPINESS.

### Some Trained Nurses' Views.

*To the Editor of* The Hospital.

Sir,—Ierne's article in last week's Hospital contains some interesting statements concerning Ireland. In the writer's anxiety to secure fair-play for the rank-and-file nurse it seems to me that some rather important points are overlooked. I have never been a matron nor held a sister's post, and it is therefore as a rank-and-file nurse that I venture to tell something of my experience. In passing, I might remark that I have myself been a patient in the private nursing home to which Ierne refers. At the time I knew as little of nursing as the gentleman whose remarks are cited, but I would like to say that during a period of three months I never heard nor saw any sign of tyranny or mortal terror amongst the nurses, who were a very happy lot and better paid than in any other nursing home in Ireland of which I know. In the light of my subsequent experience I can also truthfully affirm that they had a better time than falls to the lot of the majority. If this had not been the case it is not likely that I should ever have found myself a stiff and awkward pro. wrestling with an unfamiliar broom.

No human being is ideal, and it is as unreasonable to expect perfection in a matron or a sister as in a clergyman. But I have worked under every variety of authority and know what it means. The nurse who cares at all about her work is happiest under an authority who works herself, insists on work being well done, and is at the same time considerate. But—and I have questioned many a fellow-nurse on this subject to see if their experience would tally with mine, and always had the same reply—every decent nurse would rather work under the strictest of disciplinarians of whom she was afraid than under a slack authority who overlooked carelessness and did not keep her up to the mark. I have always noticed that the nurses who were unhappy in the strict wards were the lazy ones, who grumbled because they got no chance of scamping their work. Human nature, when young, is often overwhelmed with a desire to scamp its work, and the nurse who thinks of anything else besides her off-duty time, is in her secret soul thankful for the discipline which helps her to overcome that desire and to maintain the high standard of which she knows she is capable.

The rank-and-file nurses are very grateful for your championship, which has doubtless done much to educate public opinion, but a great many of us think that Ierne sometimes lays the blame on the wrong shoulders, and secretly agree that we would rather sweat in the wards to the end of our nursing days than be a matron—she gets all the kicks and precious few of the ha'pence.

I should be obliged if you would kindly publish this letter.—Yours, etc., Vera Matheson.

